# Magnesium isoglycyrrhizinate shows hepatoprotective effects in a cyclophosphamide-induced model of hepatic injury

**DOI:** 10.18632/oncotarget.16629

**Published:** 2017-03-28

**Authors:** Wenjiao Jiang, Jingyan Liu, Peijin Li, Qianfeng Lu, Xue Pei, Yilin Sun, Guangji Wang, Kun Hao

**Affiliations:** ^1^ Key Laboratory of Drug Metabolism and Pharmacokinetics, China Pharmaceutical University, Nanjing 210009, China; ^2^ Department of Physiology and Pharmacology, China Pharmaceutical University, Nanjing 210009, China

**Keywords:** magnesium isoglycyrrhizinate, cyclophosphamide, hepatic injury

## Abstract

The purpose of the current study was to investigate the effect of Magnesium Isoglycyrrhizinate (GM) on cyclophosphamide (CP)-induced hepatic injury *in vivo* and *in vitro*. The results demonstrated that GM exerted a protective effect on CP-induced acute liver injury, as evidenced by the alleviations of hepatic pathological damage and serum transaminase activities. Meantime, GM attenuated serum and HepG2 cell supernatant levels of TNF-α, IL-6, IL-1β, SOD and MDA. Western blot results presented that GM down-regulated the expressions of the microtubule associated protein 1A/1B-light chain 3 (LC3), Lysosome associated membrane protein-1 (LAMP-1), p-phosphatidylinositol 3-kinase (PI3K), p-protein Kinase B(Akt), p-mechanistic target of rapamycin(mTOR), p-ribosomal protein S6 kinase 70 kDa (p70S6K), p-4E binding protein 1(4EBP1), p- inhibitor of NF-κB(IκB)α and p-nuclear factor kappa B(NF-κB)p65 in CP-stimulated hepatic tissue and HepG2 cells. Taken together, our results suggested that GM showed beneficial effect on CP-induced liver injury through NF-κB-mediated inflammation and PI3K/Akt/mTOR/p70S6K/4EBP1 axis-mediated autophagy *in vivo* and *in vitro*.

## INTRODUCTION

Cyclophosphamide (CP) is one of the universally used antineoplastic drugs for its therapeutic effects against various types of tumors, multiple sclerosis, rheumatoid arthritis and systemic lupus erythematosus. Despite broad spectrum of application, CP is still clinically restricted because of the adverse effects and toxicities including vomiting, nausea, alopecia, carcinogenicity nephrotoxicity, urotoxicity, cardiotoxicity, teratogenicity and immunotoxicity and hepatotoxicity [1]. The biochemical pathogenesis of CP-induced toxicities is considered to be implicated with inductions of inflammatory cascade and oxidative stress through generations of inflammatory cytokines and free radicals in tissues [2].

Autophagy, a special catabolic pathway, is a dynamic diverse process including the formation of autophagosomes, fusion of the autophagosome with the lysosome to turn into the autolysosome, as well as consequently the degradation of cytoplasmic organelles or cytosolic components in the autolysosome [3]. During the response to some fluctuations beyond a certain threshold which was considered as ‘stress’, cells would take diverse action of stress response pathways to adapt their metabolism and protect themselves against potential damage to maintain survival. One of the key pathways induced by stress is macroautophagy, which was referred to simply as “autophagy” [4]. Once autophagy, cells produced autophagosomes which was double-membraned vesicles to sequester organelles and proteins for delivery to the lysosome followed by degradation in the lysosome [5]. The core pathway of mammalian autophagy begins with the formation of a phagophore and involves several molecular components, such as Beclin 1 pathway and PI3K/Akt/mTOR pathway. The present study was aimed to investigate the effect of GM on the regulation of PI3K/Akt/mTOR pathway.

As a magnesium salt of 18α-GA stereoisomer, Magnesium Isoglycyrrhizinate (GM) was found to exert anti-inflammatory and hepatoprotective activities on multiplex liver diseases. Chunfeng Xie *et al*. found Magnesium Isoglycyrrhizinate inhibited PLA2 activation and inflammatory lipid mediators in RAW264.7 macrophages stimulated with lipopolysaccharides (LPS) [6]. Former study also elucidated that Magnesium Isoglycyrrhizinate ameliorated ischemia/reperfusion-induced liver injury through enhancing PI3K/Akt activity in human hepatic L02 cells [7]. Magnesium Isoglycyrrhizinate was demonstrated to attenuate free fatty acid-induced lipotoxicity by reducing mitochondrial damage, which indicated a promising therapeutic intervention for nonalcoholic fatty liver disease [8]. However, it has never been proposed in previous studies about the effects of Magnesium Isoglycyrrhizinate on CP-induced liver injury which remains one of adverse consequence in cancer patients.

Although there are lots of reports about the health benefits of GM, there has been few reports evaluating the effect of GM on CP-induced hepatotoxicity. Considering the essential of liver in maintaining the body's metabolic homeostasis, our study was aimed to explore the pharmacological effects of GM for the CP-induced liver toxicity. The results of the present research might provide a complementary strategy for dealing with the hepatic toxicity of CP and other chemotherapeutic compound.

## RESULTS

### Effects of GM on liver functional enzymes in serum and cell supernatant

**Figure 1:**
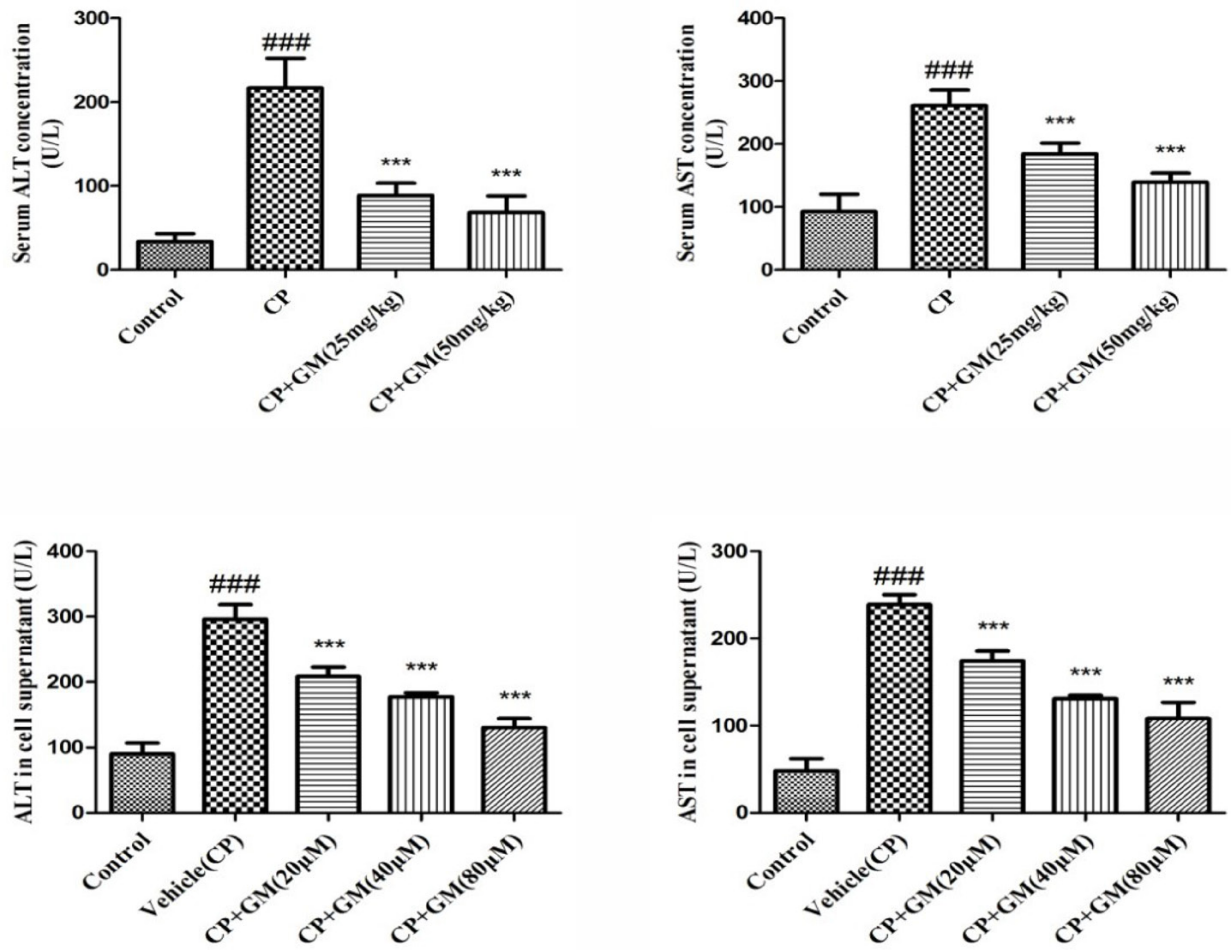
The effects of GM on activities of ALT and AST in serum and cell supernatant The data was presented as means ± SDs. Compared with control: ^#^*P* < 0.05, ^##^*P* < 0.01, ^###^*P* < 0.001. Compared with CP: *P < 0.05, **P < 0.01, ***P < 0.001.

Activities of ALT and AST in serum and cell supernatant were determined to examine the degree of hepatocyte damage. As revealed in Figure 1, AST and ALT levels of CP group in both serum and cell supernatant maintained the higher concentration compared with those of control group, suggesting hepatotoxicity in mice and cells were caused by administration of CP. However, animals or HepG2 cells treated with GM exhibited a marked reduction of ALT and AST activities. And more significant reversion could be observed after treatment with a higher dose of GM *in vivo* or *in vitro*, which suggested that GM could ameliorate acute liver injury triggered by CP challenge.

### Effect of GM on cell viability

**Figure 2:**
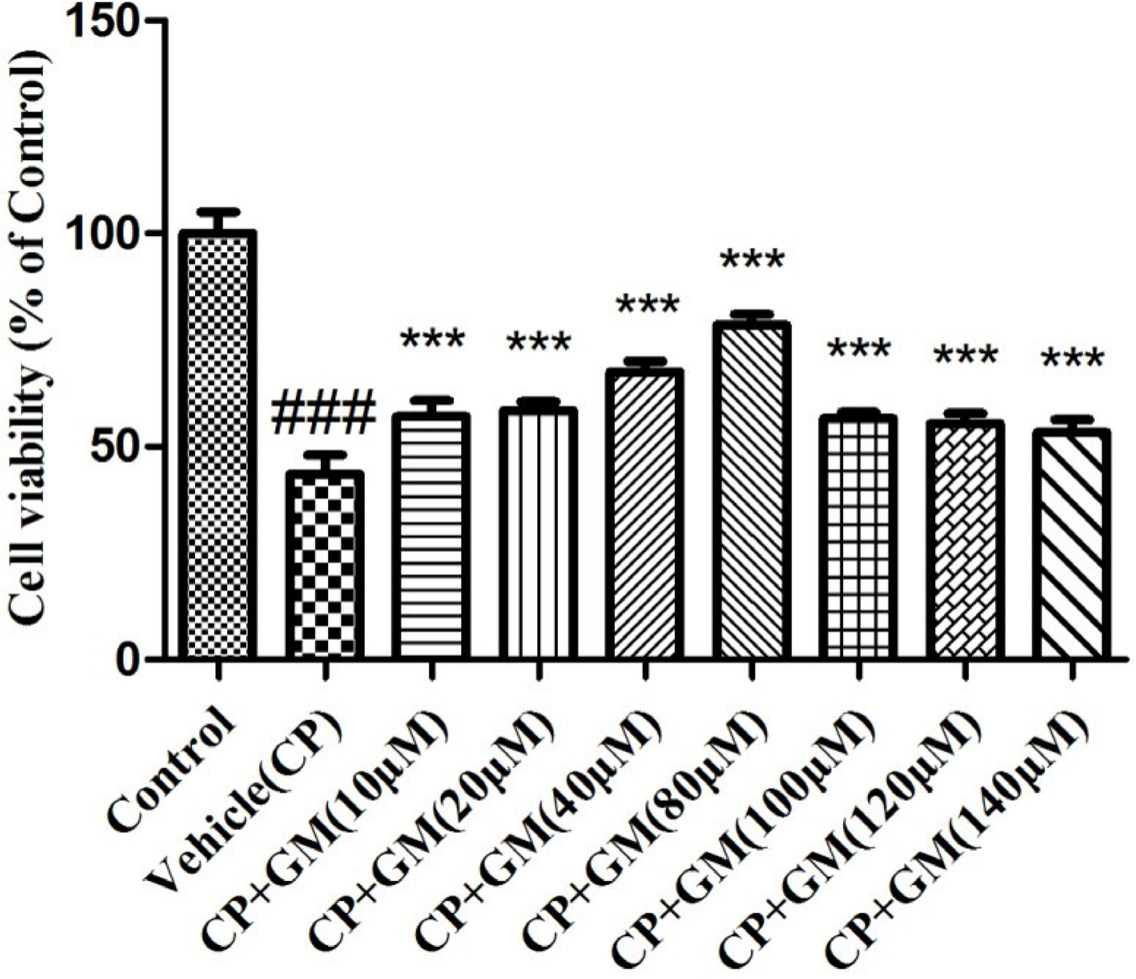
The effects of GM on cell viability using MTT assay The data was presented as means ± SDs. Compared with control: ^#^*P* < 0.05, ^##^*P* < 0.01, ^###^*P* < 0.001. Compared with CP: *P < 0.05, **P < 0.01, ***P < 0.001.

The result of the MTT assay revealed the cell survival rate in response to the administration of CP and GM. After exposure to CP(2.5 μg/ml), the cell viability of HepG2 cells in vehicle(CP) group was declined to 43.56% in contrast with that in control group. Whereas, dose-dependent ascensions in HepG2 cell viability was found after the treatment with multiple concentrations of GM (10, 20, 40 and 80 μM). It should be noted that administration of GM (100, 120 and 140 μM) displayed similar degree of inhibition in cell viability (approximately 55% of control), which belowed the viability levels of GM (80 μM). Data above demonstrated that GM exerted protective effects on HepG2 cells viability loss stimulated by CP (Figure 2).

### Effects of GM on oxidative stress in serum, liver tissue and cell supernatant

Lipid peroxidation in serum, liver tissue and cell supernatant were determined by detecting alterations in the productions of SOD and MDA. As expected, the current study observed remarkable increase of MDA as well as decrease of SOD in serum and liver tissues, while GM (25, 50 mg/kg) treatment exhibited a marked reversion of SOD, MDA as compared with those in model group. Meantime, it was noteworthy that somehow GM (50 mg/kg) administration seemed to possess a more unfavourable potency than GM (25 mg/kg) treatment in terms of the serum and hepatic levels of SOD, MDA.

**Figure 3:**
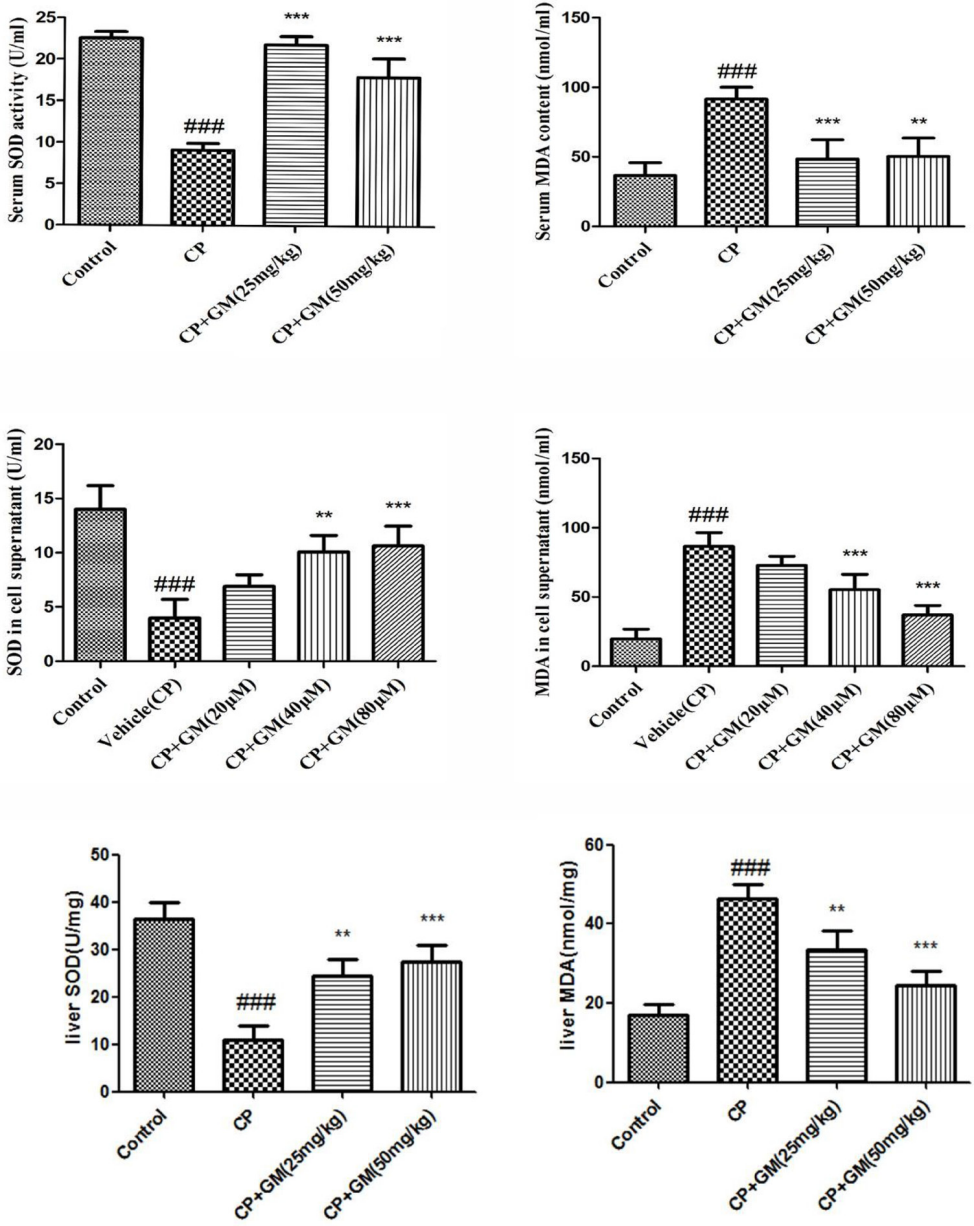
The effects of GM on the levels of SOD and MDA in serum and HepG2 cell supernatant The data was presented as means ± SDs. Compared with control: ^#^*P* < 0.05, ^##^*P* < 0.01, ^###^*P* < 0.001. Compared with CP: *P < 0.05, **P < 0.01, ***P < 0.001.

The levels of SOD, MDA in GM-treated HepG2 cells were investigated in the present study. As depicted in Figure 3, the treatment of HepG2 cells with GM resulted in concentration-dependent alterations in SOD, MDA levels in cell supernatant. Treatment with 80 μM GM resulted in 2.67-fold increase of SOD relative to that of vehicle(CP) cells. Meanwhile, consistent with the SOD masurement, 80 μM GM showed a significant inhibitory effect on generation of MDA in HepG2 cells with a approximate half levels of vehicle(CP) group. (Figure 3).

### Effects of GM on cytokines in serum and cell supernatant

**Figure 4:**
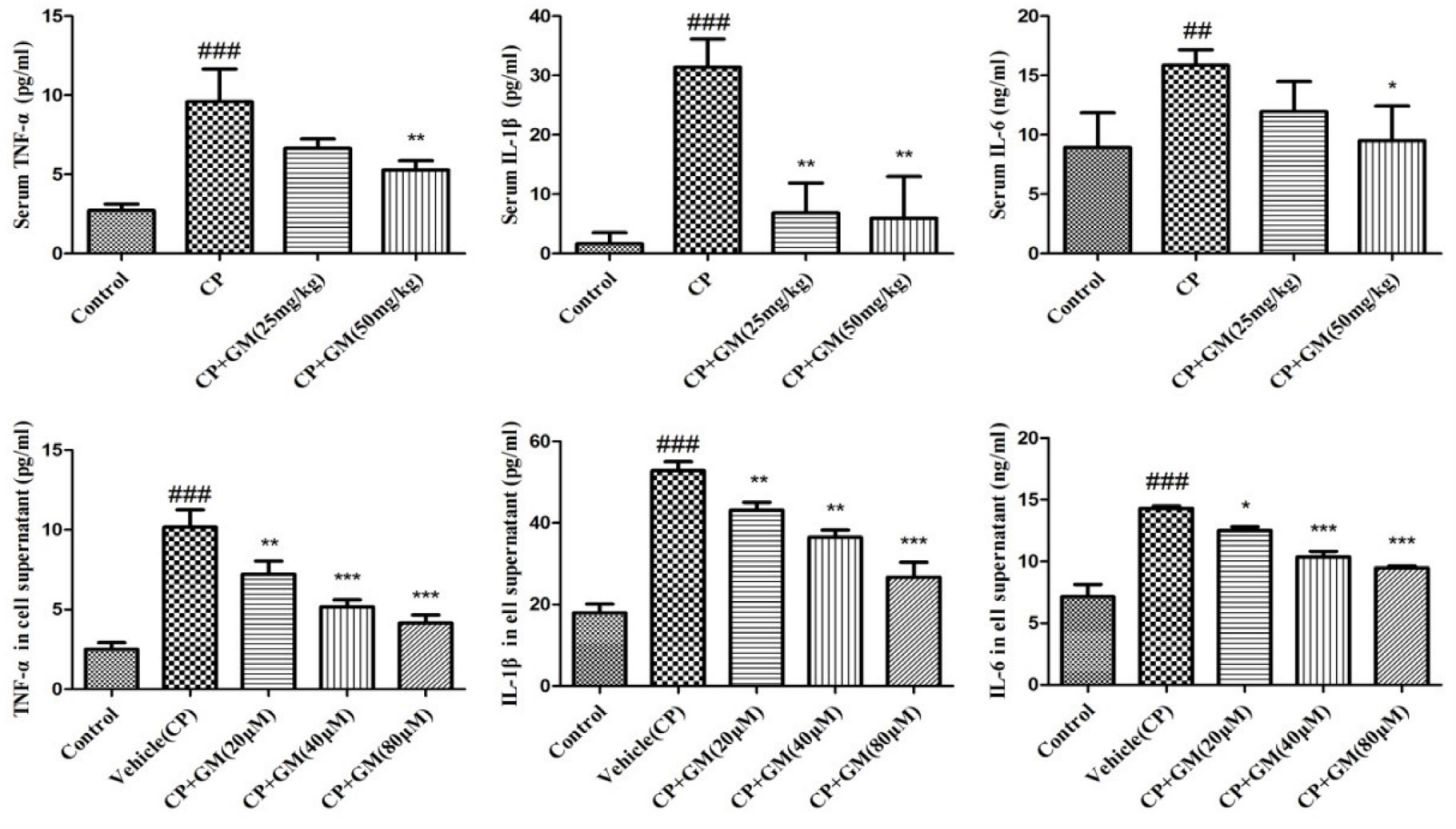
The effects of GM on the contents of IL-6, IL-1β and TNF-α in serum and HepG2 cell supernatant The data was presented as means ± SDs. Compared with control: ^#^*P* < 0.05, ^##^*P* < 0.01, ^###^*P* < 0.001. Compared with CP: *P < 0.05, **P < 0.01, ***P < 0.001.

To evaluate inflammation severity triggered by CP challenge, we investigated the productions of TNF-α, IL-1β, and IL-6 *in vivo* and *in vitro* as illustrated in Figure 4. As expected, the serum levels of IL-6, IL-1β, and TNF-α were remarkably increased in CP mice, while two doses of GM treatment showed obvious reductions compared with those in CP group, with more significant inhibition of cytokines productions in CP+GM (50 mg/kg) mice.

In addition, the contents of pro-inflammatory cytokines were significantly increased in vehicle group induced by CP, whereas these conditions were markedly attenuated by the intervention of GM (20, 40, 80 μM). These results showed that GM suppressed the release of pro-inflammatory cytokines including TNF-α, IL-1β, and IL-6 in concentrated-dependent manners in CP-stimulated HepG2 cells.

### Effects of GM on CP-induced hepatic necrosis

**Figure 5:**
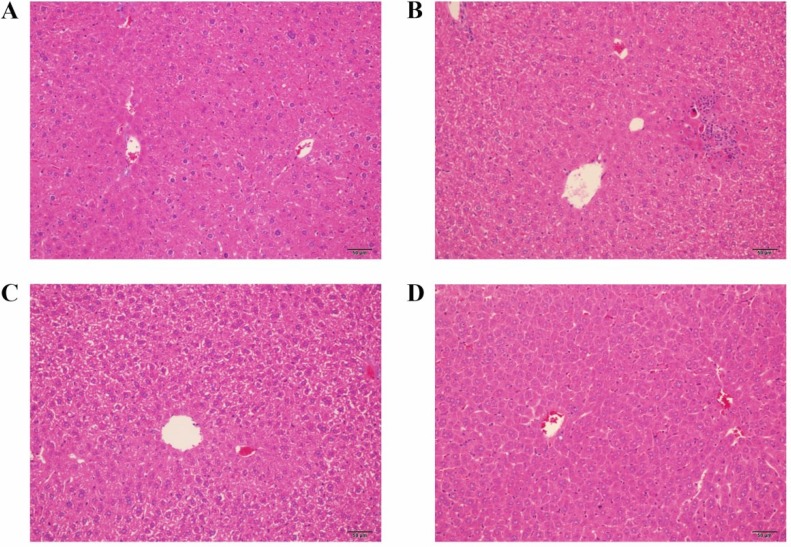
The effects of GM on histopathological alterations in hepatic tissues Data shown are representative photographs from each treatment group of mice. (**A**) the control group; (**B**) the CP group; (**C**) the CP+GM (25 mg/kg) group; (**D**) the CP+GM (50 mg/kg) group.

The protective effect of GM on CP-induced hepatic necrosis was assessed by histopathological examination, which supported the findings of biochemical analysis. Representative photomicrographs from mice liver were displayed as Figure 5 after three times exposure to CP as well as treatments with GM for consecutive six days. No appreciable histopathological alterations were investigated in the control group (Figure 5A). By contrast, striking liver injury was observed in animals from the CP group, there was obvious swelling of hepatocytes and inflammatory cell infiltration (Figure 5B). However, liver histopathological damage was significantly relieved in the CP+GM (25, 50 mg/kg)group (Figure 5C and Figure 5D), GM, especially in the 50 mg/kg group, attenuated the histopathological alterations induced by CP. And the photomicrograph of CP+GM (25 mg/kg) group exhibited minute swelling and vacuolization of hepatocytes without inflammatory cell infiltration.

### Effects of GM on expression of PI3K / Akt/ mTOR/ p70S6K/ 4EBP1/ NF-κB pathway *in vivo* and *in vitro*

**Figure 6:**
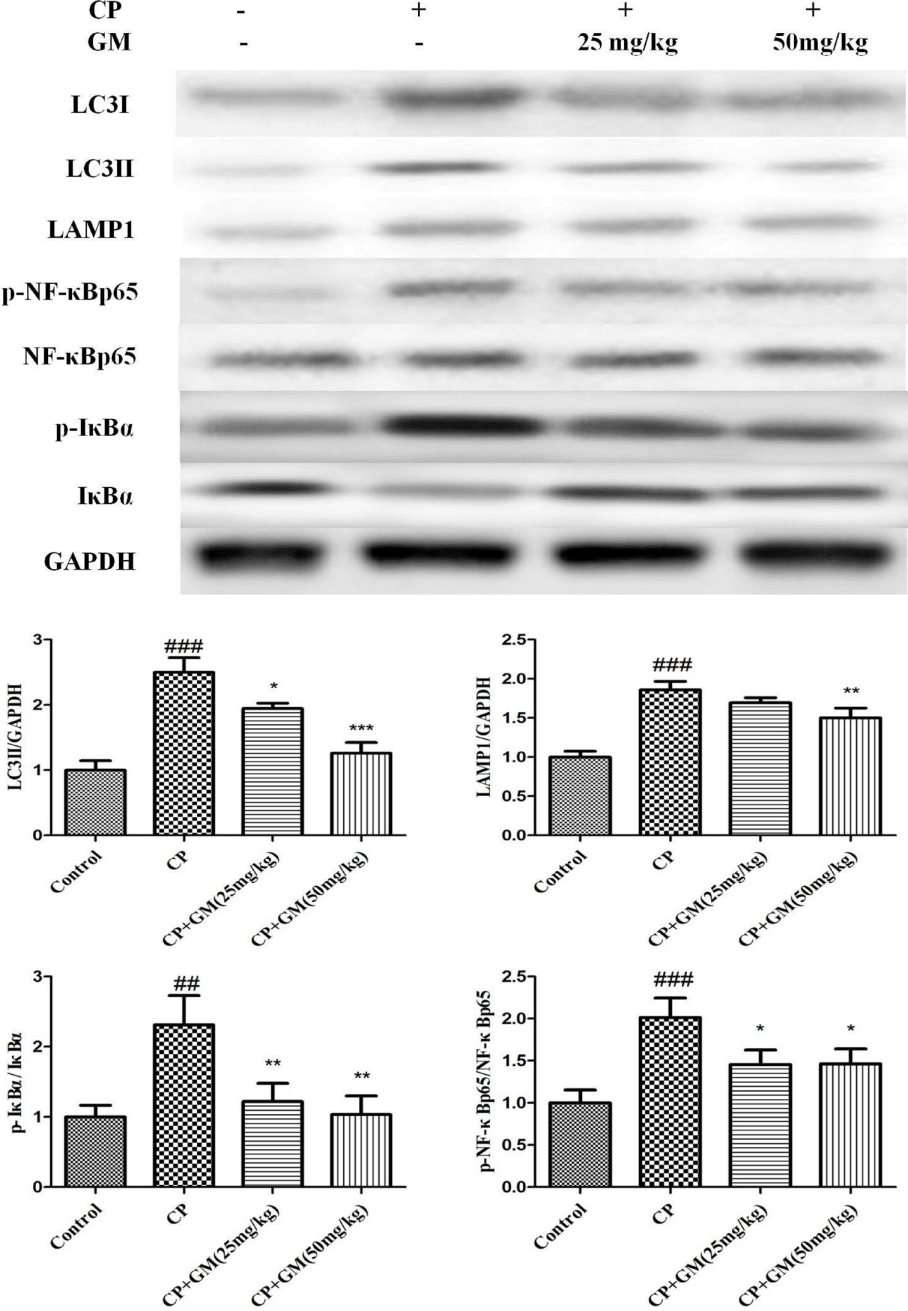

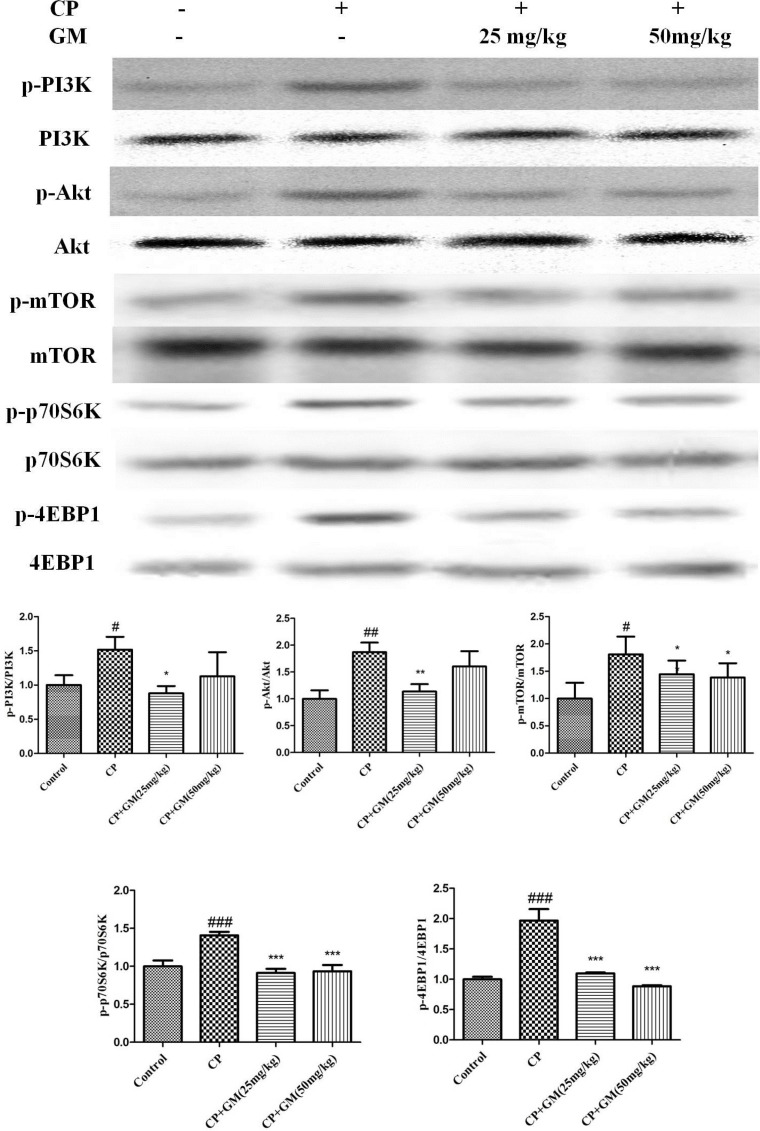
The effects of GM on the protein expressions of PI3K/Akt/mTOR/p70S6K/4EBP1/NF-κB axis in hepatic tissues Compared with control: ^#^*P* < 0.05, ^##^ *P*< 0.01, ^###^*P* < 0.001. Compared with CP: *P < 0.05, **P < 0.01, ***P <0.001.

To explore the hepatoprotective-related signaling of GM treatment, the phosphorylated and non-phosphorylated forms of the pathway components were determined respectively in hepatic tissues. As shown in Figure 6, CP-induced group had obvious up-regulated LC3I, LC3II, LAMP1, mTOR, p-p70S6K, p-4EBP1, p-p65, and p-IκBα expressions in liver tissues. Notably, GM effectively suppressed the phosphorylation of the key regulators in PI3K/Akt/mTOR and NF-κB pathways with a more obvious restore in mice treated with GM (50 mg/kg) than GM (25 mg/kg).

**Figure 7:**
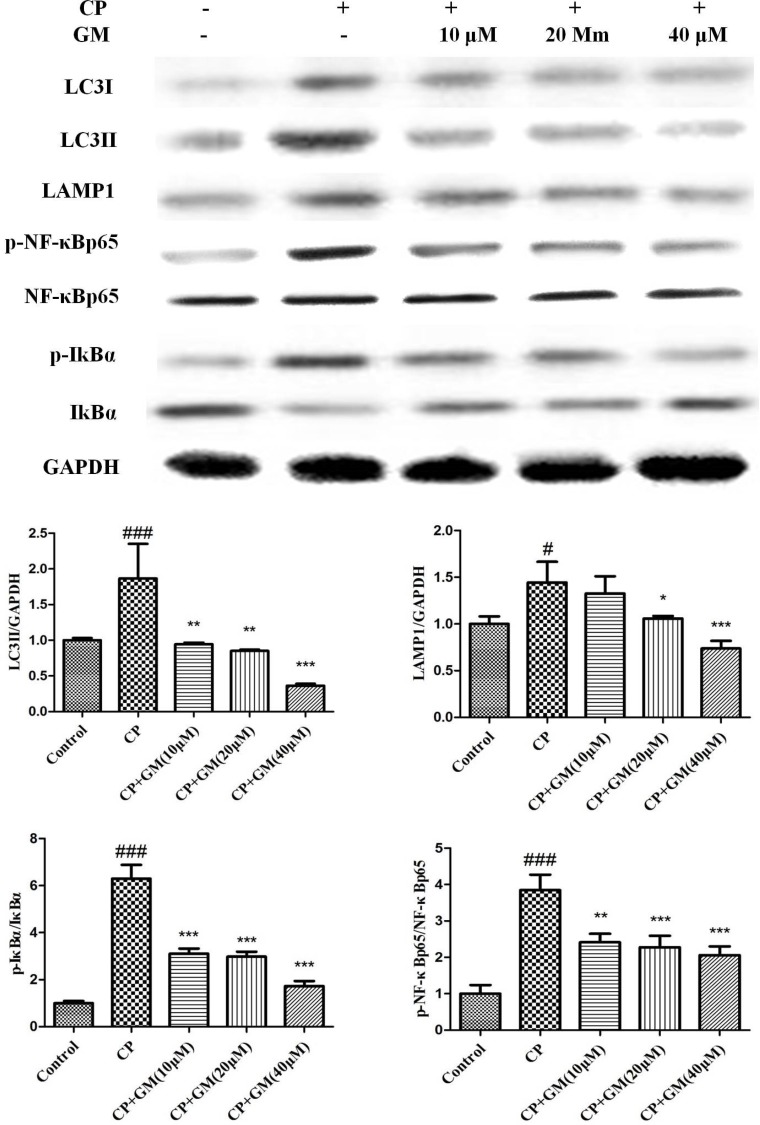

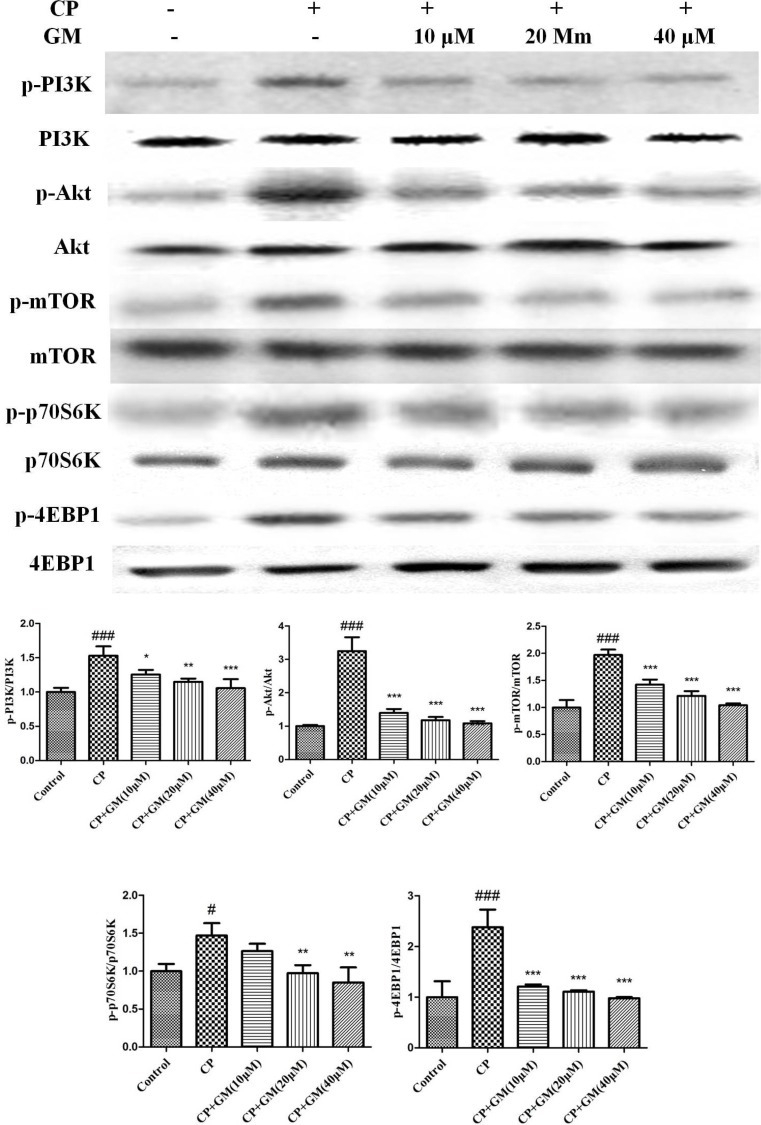
The effects of GM on the protein expressions of PI3K/Akt/mTOR/p70S6K/4EBP1/NF-κB axis in HepG2 cell supernatant Compared with control: ^#^*P* < 0.05, ^##^*P* < 0.01, ^###^*P* < 0.001. Compared with CP: *P < 0.05, **P < 0.01, ***P < 0.001.

To further elucidate the underlying mechanism of GM, we measured the expressions of mTOR and NF-κB pathway in CP-induced HepG2 cells. As illustrated in Figure 7, CP challenge significantly up-regulated the levels of LC3I, LC3II, LAMP1, mTOR, p-p70S6K, p-4EBP1, p-IκBα and p-NF-κBp65 compared with those in control cells. Nevertheless, treatment with GM remarkably blocked the phosphorylation of PI3K/Akt/mTOR/p70S6K/4EBP1/NF-κB axis in a dose-dependent manner.

## DISCUSSION

The present study was designed to demonstrate the protective effect of GM against CP-stimulated liver injury. Also, the underlying mechanisms of GM on oxidative stress, inflammatory response and autophagy signaling pathways were elucidated. High doses of CP usually results in a multiorgan lesion within several days of the administration. Cellular mechanisms of CTX-induced toxicity are considered to be mediated by the dysregulations of inflammation, free oxygen radicals (ROS) and other subsequent events [9].

AST and ALT are the reliable biochemical indices to reveal the content of early acute hepatic damage. The data proved that the administration of CP induced an obvious increase in serum transaminase levels. Furthermore, H&E examination also revealed the pathological alteration including vast areas of cellular necrosis, extensive vacuolization and inflammatory cell infiltration. The experimental findings indicated the occurrence of liver injury. On the contrary, treatment with GM recovered the hepatic function, which was supported by the amelioration of the pathological conditions and transaminase activities.

Experimental evidence displayed that oxidative stress was the important cause of cyclophosphamide hepatotoxicity. CP-stimulation accelerated lipid peroxidation and DNA damage. The oxidative effects produced by CP-exposure are associated with an accumulation of hydrogen peroxide (H_2_O_2_). Whereas SOD is responsible for detoxification of anion superoxide (O^2−^) and production of H_2_O_2_. As the end product from lipid breakdown, MDA is recognized as a reliable indicator sensitive to oxidative stress in hepatic lesion [10]. It was proposed that a single dose of CP(100 mg/kg, i.p.) conduced to oxidative stress in liver tissues of Swiss mice, evidenced by increased content of malondialdehyde (MDA) and weaken antioxidant defenses [11]. Selvakumar *et al*. elicited that CP increased MDA and hydrogen peroxide levels as well as changed SOD [12]. Our data demonstrated that GM reduced the MDA content and restored SOD activity in CP-induced hepatic injury.

A broad spectrum of studies pointed out that CP could induce the inflammatory response in a variety of organs [13]. CP was reported to cause the overproductions of various pro-inflammation cytokines including TNF-α, IL-1β and IL-6 [14]. The excessive inflammatory cytokines participate in the initiating, accelerating and perpetuating the inflammatory process during the pathogenesis of liver injury [15, 16]. The present result revealed that CP stimulation changed in the generation of several inflammatory cytokines, while this effect was evidently antagonized by GM, confirming the anti-inflammatory role of GM in CP-induced hepatic injury.

As a hot spot for recent researches, autophagy is a survival-associated mechanism for degradation of defective organelles. Recent studies have revealed the correlation between autophagy and the development of liver diseases as well as the therapeutic potential of regulated autophagy. The widely used biological marker to monitor autophagy in mammalian is the microtubule associated protein 1A/1B-light chain 3 (LC3). During autophagy, cytosolic LC3-1 is conjugated to phosphatidylethanolamine (PE) to form LC3-II. LC3-II is recruited to elongate the autophagosomal membrane, which contributes to autophagosome formation [17]. The fusion of the autophagosome and lysosome to form autolysosome requires essential molecules including LAMP1. Lysosome associated membrane protein-1 (LAMP-1), the major protein components of the lysosomal membrane, is known to be involved in lysosome biogenesis and autophagy [18]. Several signaling pathways are involved in the formation and process of autophagy, among which, the class I PI3K/Akt/mTOR signaling pathway is the best characterized. The mTOR kinase signaling pathway participates in the regulation of energy, growth signals, cell division [19]. mTOR responds to numerous stresses, while its dysregulation could result in cancer, metabolic disease and diabetes. In cells, mTOR exists as two totally different complexes in function or structure, namely mTOR complex 1 (mTORC1) and mTOR complex 2 (mTORC2) [20]. Among them, the activiation of mTOR complex 1 (mTORC1) was proved to play an important role in the process of autophagy [21]. Intriguingly, PI3K activates the phosphorylation of Akt followed by the activation of mTOR, which finally trigged the inhibition of autophagy. Thus, class I PI3K/Akt/mTOR conversely regulates autophagy, as evidenced by the up-regulation of autophagy induced by the inhibition of Akt [22]. A noteworthy insight arising from the data above was the opposite alteration in the regulation of downstream translational targets of mTORC1 (either p70S6K or 4EBP1) [23]. In the present study, the autophagy was induced by CP challenge as evidenced by the increased levels of LC3, p70S6K and 4EBP1, while GM effectively reversed these alterations *in vivo* and *in vitro*. However, the opposite alteration in the expression of PI3K/Akt/mTOR pathway indicated that the GM treatment might exhibit their inhibitory effect against autophagy through other autophagy-related approaches instead of PI3K/Akt/mTOR signaling pathway.

NF-κB signaling pathway is implicated in the regulation of multiple cellular mechanisms, such as apoptosis, autophagy, metabolism and inflammation immunity responses. Emerging studies have reported that the IKK/NF-κB signaling can promote autophagy and evoke the expression of Beclin 1 or other autophagy-related proteins. These events stimulates the initiation of autophagy, while autophagy conversely degrades IKK components as a feedback response [24–26]. Our data revealed the expression of NF-κB pathway was evoked after CP exposure, which was consistent with previous studies. However, GM effectively suppressed the phosphorylation of NF-κB pathways with a higher restore in mice treated with GM (50 mg/kg) than GM (25 mg/kg) compared with those in CP-challenged mice.

In summary, the present study revealed that Magnesium Isoglycyrrhizinate (GM) could effectively attenuate cyclophosphamide (CP)-induced liver injury *in vivo*, which was involved in the down-regulation of autophagy and NF-κB pathways. In addition, the protective effect of GM on liver injury stimulated by CP was demonstrated to be correlated with the attenuation of oxidant stress and hepatocellur inflammation. Further studies are warranted to elucidate about Magnesium Isoglycyrrhizinate.

## MATERIALS AND METHODS

### Reagents

**Figure 8:**
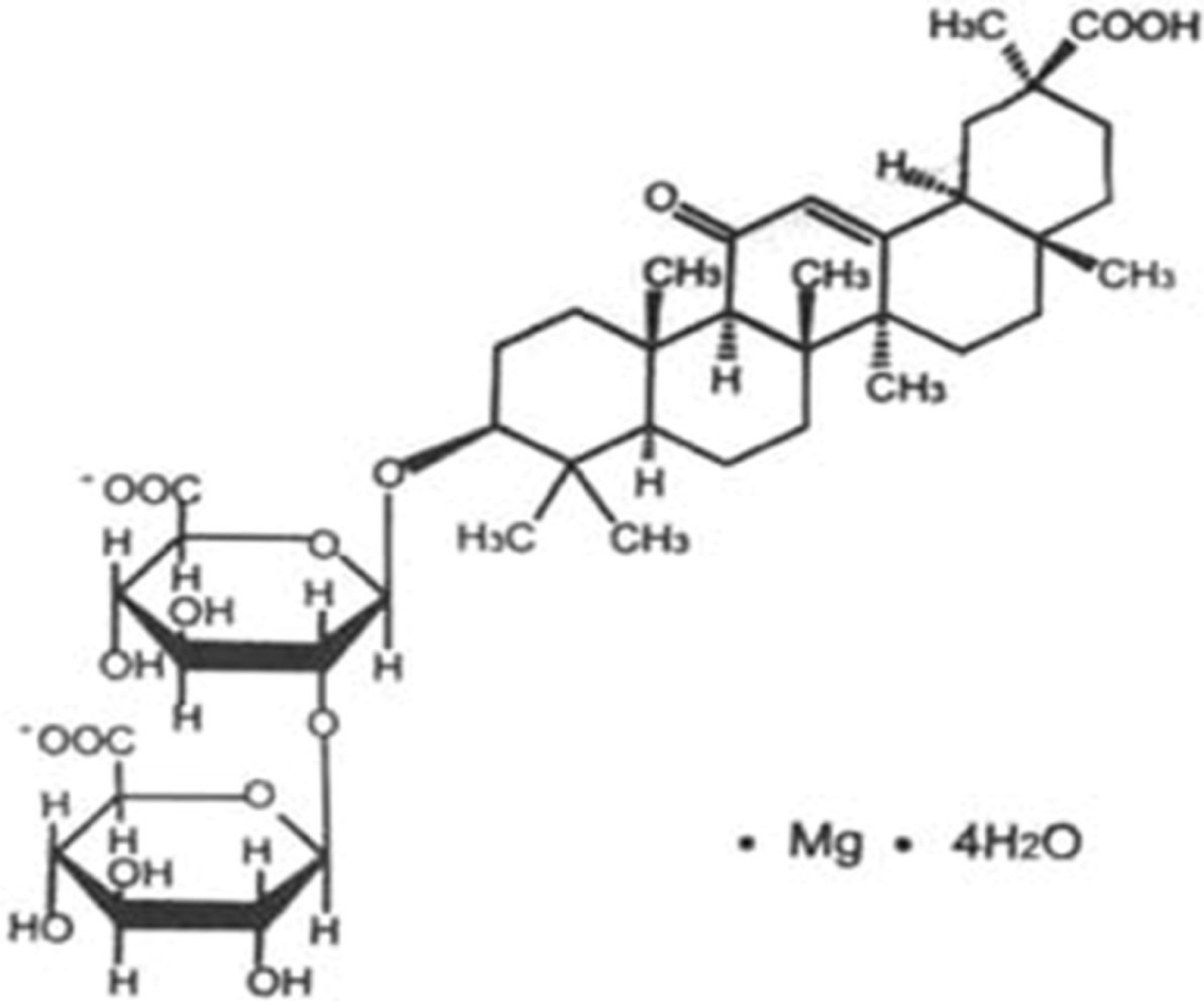
The chemical construction of GM

Magnesium Isoglycyrrhizinate (GM) injection was purchased from Chia Tai Tianqing Pharmaceutical Group Co., Ltd. The construction of Magnesium Isoglycyrrhizinate (GM) was shown in Figure 8. Cyclophosphamide (CP) was obtained from Sigma Chemical Company (St. Louis, USA). ELISA kits of tumor necrosis factor-α(TNF-α), interleukin-1β(IL-1β) and interleukin-6(IL-6) were obtained from Nanjing KeyGEN Biotech. Co. Ltd. (Nanjing, China). The alanine aminotransferase (ALT), aspartate aminotransferase (AST), superoxide Dismutase (SOD) and malondialdehyde (MDA) detection kits were obtained from Nanjing Jiancheng Bioengineering Institute (Nanjing, China). All antibodies were provided by Cell Signaling Technology (Danvers, USA).

### Animals and treatment

Balb/c mice (6 weeks old, 18–22 g) in the present experiment were supplied by B & K Universal Group Limited, Shanghai, China. The animals were allowed to acclimatize to the facilities under controlled environmental conditions (24 ± 1°C temperature; 40–50% humidity; 12 h light/dark cycle) with free access to diet and water for one week prior to animal experiment. All animal procedures were performed in accordance with the National Laboratory Animal Management Regulations and the experimental protocol was approved by the Animal Ethics Committee of China Pharmaceutical University (Nanjing, China).

Male Balb/c mice (18–22 g) were randomly divided into four groups: control group, CP group, CP + GM (25 mg/kg) group and CP + GM (50 mg/kg) group, with ten mice each group. Based on our preliminary experiments the dose of GM was decided. Protective activity of GM was evaluated with intraperitoneal treatment of multiple doses (25, 50 mg/kg/day, 6 consecutive days). The dose of CP (80 mg/kg) was selected based on earlier reports to induce liver injury [27]. For animals in the CP + GM (25 mg/kg) group and CP + GM (50 mg/kg) group, cyclophosphamide was intraperitoneally administered at a concentration of 80 mg/kg 1 h prior to GM administration at 1, 3, 5 days. The control mice received the same volume of saline. 24 hours after the final administration of GM (25, 50 mg/kg/day), blood samples were collected from their petro-orbital plexus, centrifuged at 8000 rpm for 5 min and then the serum samples were stored at −80°C for following biochemical tests. Thereafter, the liver was isolated for Hematoxylin-eosin (H&E) staining and western blotting after animals were sacrificed by ether anesthesia.

### Cell culture

Human hepatocyte-derived cell line HepG2 was purchased from the American Type Culture Collection(ATCC, Rockville, MD). The cells were maintained in Dulbecco's modified Eagle's medium(DMEM) containing 10% fetal bovine serum (Hyclone, South,America), 100 U/mL penicillin, and 100 μg/mL streptomycin (Amresco, USA) at 37°C in a 5% CO_2_ humidified incubator. Cell count was assessed by Trypan Blue exclusion before MTT assay.

### MTT assay for cell viability

Cell viability was determined by MTT-based colorimetric assay. In brief, the cells were plated in 96-well plates at an approximate density of 3000 cells per well to incubate and treated with different concentrations of GM (10, 20, 40, 80, 100, 120, 140 μM) 6 h after the addition of CP (2.5 μg/ml). Untreated cells acts as control. After incubation for 24 h, the cells were exposed to MTT (5 mg/ml) followed by incubation at 37°C for another 4 h. Subsequently, and the formazan crystals were dissolved under 150 μl DMSO after the removal of medium. The absorbance of the resulting color in each well was read using microplate spectrophotometer (Tecan, Switzerland) at a wavelength of 570 nm.

### Aminotransferase levels

The activities of ALT, AST in serum and cell supernatant were determined with commercial test kits (Nanjing Jiancheng Bioengineering Institute, China) according to the manufacturer's protocol. The analyte concentrations of ALT and AST were calculated based on the standard curve.

### Evaluation of oxidative stress

The levels of SOD and MDA in serum, liver and cell supernatant were examined using test kits derived from Nanjing Jiancheng Bioengineering Institute (Nanjing, China). All procedures were performed according to the protocol recommended by the manufacturer.

### Measurement of pro-inflammatory cytokines in serum

The concentrations of IL-6, IL-1β, and TNF-α in serum and cell supernatant were detected with ELISA Kits (KeyGEN, China) according to the manufacturer's instructions. The absorbance of each well was identified under 450 nm using a microplate spectrophotometer.

### Histopathological evaluation

The Hepatic tissue samples collected at 24 hours after the final administration of GM(25, 50 mg/kg/day) were fixed with 10% buffered formalin, embedded with paraffin and sliced at a thickness of 5 μm. Subsequently, sections stained with hematoxylin and eosin (H&E) according to the stand protocol were observed under a light microscope. The histopathological evaluation was performed by pathologists in a blinded manner.

### Western blot

The hepatic tissue and HepG2 cells treated with CP were taken to investigate the alterations of protein expressions. Hepatic tissue and cells were homogenized and lysed in a RIPA buffer (Sigma, St.Louis, MO, USA). The dissolved proteins were collected from the supernatant after centrifugation at 12,000 g for 20 min. The total protein concentrations were accomplished using protein extract kit (Thermo) according to the manufacturer's protocol. Equal amount of protein was subjected to SDS-PAGE and transferred to polyvinylidene fluoride membranes (Millipore Corporation, Boston, MA, USA). The membranes were blocked using 5% nonfat milk for 2 h at room temperature and incubated overnight with primary antibodies at 4°C followed by treatment with a horseradish peroxidase-conjugated secondary anti-rabbit antibody. A gel imaging system (ChemiScope 2850, Clinx Science Instruments Co, Ltd, Shanghai, China) was employed to visualize the immunoreactivity. The relative optical densities of specific proteins were identified with a ChemiScope analysis program.

### Statistical analysis

The data were displayed as mean values ± SDs. Differences between groups were produced by one-way ANOVA with Tukey multiple comparison test, with *p* < 0.05 considered as significant.

## References

[1] Bhattacharjee A, Basu A, Biswas J, Bhattacharya S (2015). Nano-Se attenuates cyclophosphamide-induced pulmonary injury through modulation of oxidative stress and DNA damage in Swiss albino mice. Mol Cell Biochem.

[2] Song Y, Zhang C, Wang C, Zhao L, Wang Z, Dai Z, Lin S, Kang H, Ma X (2016). Ferulic Acid against Cyclophosphamide-Induced Heart Toxicity in Mice by Inhibiting NF-κB Pathway. Evid Based Complement Alternat Med.

[3] Zhang XJ, Chen S, Huang KX, Le WD (2013). Why should autophagic flux be assessed?. Acta Pharmacol Sin.

[4] Kroemer G, Mariño G, Levine B (2010). Autophagy and the integrated stress response. Mol Cell.

[5] He C, Klionsky DJ (2009). Regulation mechanisms and signaling pathways of autophagy. Annu Rev Genet.

[6] Xie C, Li X, Wu J, Liang Z, Deng F, Xie W, Zhu M, Zhu J, Zhu W, Geng S (2015). Anti-inflammatory activity of magnesium isoglycyrrhizinate through inhibition of phospholipase A2/arachidonic acid pathway. Inflammation.

[7] Huang X, Qin J, Lu S (2014). Magnesium isoglycyrrhizinate protects hepatic L02 cells from ischemia/reperfusion induced injury. Int J Clin Exp Pathol.

[8] Cheng Y, Zhang J, Shang J, Zhang L (2009). Prevention of free fatty acid-induced hepatic lipotoxicity in HepG2 cells by magnesium isoglycyrrhizinate *in vitro*. Pharmacology.

[9] Sulkowska M, Sulkowski S, Skrzydlewska E (1999). The effect of pentoxifylline on ultrastructure and antioxidant potential during cyclophosphamide-induced liver injury. J Submicrosc Cytol Pathol.

[10] Jiang W, Zhou R, Li P, Sun Y, Lu Q, Qiu Y, Wang J, Liu J, Hao K, Ding X (2016). Protective effect of chrysophanol on LPS/d-GalN-induced hepatic injury through the RIP140/NF-κB pathway. RSC Adv.

[11] Tripathi D, Jena G (2009). Intervention of astaxanthin against cyclophosphamide-induced oxidative stress and DNA damage: a study in mice. Chem Biol Interact.

[12] Selvakumar E, Prahalathan C, Mythili Y, Varalakshmi P (2005). Beneficial effects of DL-alpha-lipoic acid on cyclophosphamide-induced oxidative stress in mitochondrial fractions of rat testis. Chem Biol Interact.

[13] Matar P, Rozados VR, Gervasoni SI, Scharovsky GO (2002). Th2/Th1 switch induced by a single low dose of cyclophosphamide in a rat metastatic lymphoma model. Cancer Immunol Immunother.

[14] Zoheir KMA, Harisa GI, Abo-Salem OM, Ahmad SF (2015). Honey bee is a potential antioxidant against cyclophosphamide-induced genotoxicity in albino male mice. Pak J Pharm Sci.

[15] Chen T, Gao J, Xiang P, Chen Y, Ji J, Xie P, Wu H, Xiao W, Wei Y, Wang S (2015). Protective effect of platycodin D on liver injury in alloxan-induced diabetic mice via regulation of Treg/Th17 balance. Int Immunopharmacol.

[16] Wang Q, Wen R, Lin Q, Wang N, Lu P, Zhu X (2015). Wogonoside Shows Antifibrotic Effects in an Experimental Regression Model of Hepatic Fibrosis. Dig Dis Sci.

[17] Tanida I, Ueno T, Kominami E (2008). LC3 and Autophagy. Autophagosome and Phagosome.

[18] Eskelinen EL (2006). Roles of LAMP-1 and LAMP-2 in lysosome biogenesis and autophagy. Mol Aspects Med.

[19] Zoncu R, Efeyan A, Sabatini DM (2011). mTOR: from growth signal integration to cancer, diabetes and ageing. Nat Rev Mol Cell Biol.

[20] Hara K, Maruki Y, Long X, Yoshino Ki, Oshiro N, Hidayat S, Tokunaga C, Avruch J, Yonezawa K (2002). Raptor, a binding partner of target of rapamycin (TOR), mediates TOR action. Cell.

[21] Inoki K, Li Y, Zhu T, Wu J, Guan KL (2002). TSC2 is phosphorylated and inhibited by Akt and suppresses mTOR signalling. Nat Cell Biol.

[22] Shintani T, Klionsky DJ (2004). Autophagy in health and disease: a double-edged sword. Science.

[23] Imai Y, Gehrke S, Wang HQ, Takahashi R, Hasegawa K, Oota E, Lu B (2008). Phosphorylation of 4E-BP by LRRK2 affects the maintenance of dopaminergic neurons in Drosophila. EMBO J.

[24] Nivon M, Richet E, Codogno P, Arrigo AP, Kretz-Remy C (2009). Autophagy activation by NFκB is essential for cell survival after heat shock. Autophagy.

[25] Nivon M, Abou-Samra M, Richet E, Guyot B, Arrigo AP, Kretz-Remy C (2012). NF-κB regulates protein quality control after heat stress through modulation of the BAG3–HspB8 complex. J Cell Sci.

[26] Copetti T, Bertoli C, Dalla E, Demarchi F, Schneider C (2009). p65/RelA modulates BECN1 transcription and autophagy. Mol Cell Biol.

[27] Xu W, Zhou L, Ma X, Chen Y, Qin B, Zhai X, You S (2011). Therapeutic effects of combination of paeoniflorin and albiflorin from Paeonia radix on radiation and chemotherapy-induced myelosuppression in mice and rabbits. Asian Pac J Cancer Prev.

